# Perceptions and beliefs of public policymakers in a Southern European city

**DOI:** 10.1186/s12939-015-0143-5

**Published:** 2015-02-12

**Authors:** Joana Morrison, Mariona Pons-Vigués, Elia Díez, Maria Isabel Pasarin, Sergio Salas-Nicás, Carme Borrell

**Affiliations:** Department of Epidemiology and Public Health, University College London, London, UK; CIBER de Epidemiología y Salud Pública (CIBERESP), Barcelona, Spain; Agència de Salut Pública de Barcelona, Barcelona, Spain; Institut Universitari d’Investigació en Atenció Primària Jordi Gol (IDIAP Jordi Gol), Barcelona, Spain; Universitat de Girona, Girona, Spain; Institut d’Investigació Biomèdica Sant Pau (IIB Sant Pau), Barcelona, Spain; Universitat Pompeu Fabra, Barcelona, Spain

**Keywords:** Health inequalities, Public policies, Urban health, Policymaker, Perception, Knowledge, Qualitative research

## Abstract

**Introduction:**

Socio-economic inequalities in health are large in urban areas; however, local municipal governments may plan, manage and provide services and policies which can reduce these. The objective of this study was to describe the beliefs and perceptions of public policymakers in a European city, Barcelona. They are the key actors in designing and implementing urban public policies.

**Methods:**

A qualitative research study describing policymakers’ beliefs on health inequalities. The study population were twelve policymakers. These were politicians or officers from the city council. Informant profiles were selected using a theoretical sample. Semi-structured individual interviews were performed to collect the data and a thematic content analysis was carried out.

**Results:**

Politicians were aware of health inequalities in their city and identified diverse social causes. They viewed reducing inequalities as a priority for the city’s government. Officers were less knowledgeable and described less efforts in addressing health inequalities. It was stated by some that reducing inequalities in non-health sectors helped to reduce health inequalities indirectly and there was some collaboration between two sectors. The most frequent barriers encountered when implementing policies were funding and the cities’ limited authority.

**Conclusions:**

Officers and policymakers had different levels of awareness and access to information on health and its socials determinants. Officers referred to specific causes of health inequalities and policies which related to their sectors and politicians were more familiar with upstream determinants and policies. Some participants explained that policies and programmes needed to be evaluated and very little intersectoral action was said to be carried out. More efforts should be made to provide all policymakers with information on the social determinants of health inequalities. Research on health inequalities and policy should engage with policymakers and promote health as a cross cutting issue in the city council in liaison with the third sector.

## Introduction

Cities around the world, and specifically in Europe, have distinctive features such as high population density, social diversity and a wide range of services and facilities [1], for example: specialised health care centres and social networks and community organizations [2]. However, socio-economic inequalities in health are greater in urban than in rural settings [3]. In cities, inequalities in health affect-most predominantly-disadvantaged populations living in inner city areas or in the less serviced peripheries [4]. Nevertheless, municipal governments have substantial authority over a diversity of determinants of health inequalities [5]. Sectoral and intersectoral policies allow addressing urban inequities even though the level of competences varies between countries [6,7].

Social health inequalities have been widely documented throughout Europe [8]. Most studies compare social inequalities in health within or among countries-usually with a quantitative approach-but do not focus on cities or urban populations [9]. Knowledge regarding how policymakers from local levels of government perceive the subject is still scarce [10,11] and the few relevant studies in this field have been carried out outside Europe [12-14]. To understand the policymaking process in municipal governments, it is important to comprehensively capture the beliefs and perceptions of decision makers, as they play a key role in the non-linear, fast paced policymaking process [11,15]. This research may also provide a deeper understanding on how priorities are set in the agenda in cities’ governments [16].

This study forms part of the ‘Socioeconomic inequalities in mortality: evidence and policies in cities of Europe’ (INEQ-Cities) project, funded by DG-SANCO, European Commission [17]. The European Commission funded INEQ-Cities project has advanced the research in this area [18,19]. The project is made up of two main components: an analysis of mortality inequalities in small areas in 16 European cities and an exploratory description of policies and interventions delivered to address health inequalities in the participant cities. The second component includes a comparison of health policy documents [20], a scoping review of interventions [21], the view of managers delivering municipal interventions to address health inequalities [22] and policymaker’s perceptions on health inequalities and policies to reduce them in 13 European cities [23]. Our study is complementary to the later as it analyses the discourse of policymakers from different municipal sectors in a principal European city. Accordingly, it uses qualitative methodology to explore in-depth the following research questions: What perceptions and beliefs do policymakers have regarding health inequalities and their causes, which are political institutions’ priorities regarding inequalities in health and sectors involved? Furthermore, do social actors participate in policy making processes and which barriers and opportunities have policymakers encountered, in addition to what kind of information is available on health inequalities? The objective of this study was to describe the perceptions and beliefs of public policymakers regarding social inequalities in health and policies to reduce these in Barcelona.

## Methods

### Methodological development

This is a descriptive and exploratory qualitative study carried out in Barcelona in 2010–2011. It was performed using qualitative methodology to capture the meanings attributed by policymakers to health inequalities, based on their own experiences [24].

### Participants and sampling techniques

The study population consisted of public policymakers working in the city of Barcelona during the research period, divided into two groups: a) non-elected officers working in the council of Barcelona holding the highest ranking positions, with decision making responsibilities (officers, from now on) and b) elected politicians or aldermen/women from the city council (politicians). Participants were selected through a theoretical sample which defined the informant’s profiles to represent the discursive variants of the city’s governmental sectors (Table 1) which influence the determinants of health [5,25]. Furthermore, a member of the opposition party was also selected to include a discourse outside the coalition government. During the study period, Barcelona was governed by a social-democrat left wing and green party coalition: Partit dels Socialistes de Catalunya, (PSC-PSOE), Initiativa per Catalunya Verds (ICV) and Esquerra Unida i Alternativa (EUiA). The three main opposing parties were: a left-wing independence party; Esquerra Republicana de Catalunya (ERC), Convergència i Unió (CiU) and Partit Popular de Catalunya (PPC)*.* The latter-two being right-wing conservative parties*.* Key informants who had worked in the city council for a long period of time helped identify possible informants and, in specific cases, they liaised with them directly. One of the public health informants worked in the same institution as the authors in different premises, however and there had been very little previous contact between the interviewer and the informant. The sample consisted of twelve participants; Table 1 shows informants according to sector, position and sex.

**Table 1:** **Informant characteristics according to sector, function and sex** ^1^

**Variables to define profiles**	**Categories**	**Sex**		**Number of people**
		**Man**	**Woman**	
Sector	Public health	2	1	3
	Health care services	1	0	1
	Social welfare	1	0	1
	Social services	1	0	1
	Employment	0	1	1
	Education	0	1	1
	Urban planning and Housing	0	2	2
	Environment	0	1	1
	Structural Funds	1	0	1
Position	Politician	2	2	4
	Officer	4	4	8

^1^The information appears according to sector, position and sex instead of by informant to avoid any possibility of identification.

### Data collection

Semi-structured individual interviews were carried out following an interview guide (please see the interview guide) based on the protocol of the INEQ-Cities project and developed in consensus with all European partners. Two pilot interviews were performed in order to test the guide. The interview guide was sent to the interviewees who requested it before accepting to participate in the interview. One interviewer (JM) performed ten individual interviews and one interview with two informants (urban planning) between April 2010 and May 2011 at the participants’ workplace.

## Interview topic guide

### Topics

- *Can you explain your point of view on health inequalities in Barcelona?*
- *Which do you consider are the causes of these health inequalities?*
- *Is tackling health inequalities a priority in Barcelona?*
- *Do you have periodic information on health inequalities and policies designed to reduce them?*
- *Are there policies aimed at reducing health inequalities in Barcelona? Could you name and describe them?*
- *Do these policies cover different areas?*
- *Were these policies designed with the participation of different social agents?*
- *Sometimes some opportunities arise which may enable the implementation of interventions or policies. Please, can you provide any experience or thoughts about this?*
- *Which barriers do you face when reducing health inequalities?Do you know of policies funded with European structural funds?*

### Processing and analysis of information

One researcher (JM) transcribed the interviews and two researchers (JM, MPV) performed a thematic content analysis with the support of the Atlas.Ti qualitative data analysis programme [26]. The interviews were coded using emerging and pre-established codes, based on the research questions, and then grouped into larger analysis categories. The analysis was an iterative process and both coders discussed the minor discrepancies and resolved them together until reaching a consensus. Finally, results were contrasted with the original transcripts by both analysts. Draft versions of the manuscript were written in English. Finally the interviewer sent the results to the informants who participated in the study, as part of the validation process, and no objections were made [27].

### Ethical considerations

Informed consent was obtained through verbal means and the information was anonymised and confidential. Furthermore, no participants received a salary or reward as participation was completely voluntary. The study received formal ethical approval by the Hospital del Mar de Barcelona Research Ethics Committee.

## Results

The results have been structured under subheadings following the topics listed in the interview guide, grouped according to an emerging outline. We have described and illustrated differences in the discourses between informants working in the health and non-health sectors as well as officers and politicians. Further verbatim can be found in Table 2.

**Table 2:** **Additional verbatim following the structure of the results section**

**HEALTH INEQUALITIES**	**Informant**
**Awareness and beliefs on health inequalities**	
*“Therefore I think that Barcelona is articulated by old inequalities which have not been resolved and emerging realities which entail new related risks and social exclusion, which affect health.”*	Welfare politician
*“I think that the quality of life of people with higher purchasing capacities is different. I think that in life people accumulate factors which make their health more precarious.”*	Employment officer
**Causes of health inequalities**	
*“Gender inequalities, for example, have a very important impact on health inequalities…and here I’m referring to education inequalities, health inequalities and income inequalities along with material possessions and environmental matters.*”	Welfare politician
*“The lack of information, the lack of access to basic cultural knowledge… it causes people to be outside the inclusion mechanisms and it affects health.”*	Environmental politician
**POLICIES TO REDUCE HEALTH INEQUALITIES**	
*“I consider that the educational system palliates health inequalities because we have a whole offer of education and that is one of strong policies of the consortium; we create a protective surrounding for the student.”*	Education, officer
“A lot has to do with city health care services to be accessible and wide spread and to correct inequalities.”	Public health officer
**THE ROLE OF THE CITY GOVERNMENT IN REDUCING HEALTH INEQUALITIES**	
**Priorities of the city government**	
*“What a public administration aims for is to build a more just, equal, egalitarian and supportive city and, regarding health, it sets out to improve its citizen’s health.”*	Public health politician
*“Reducing health inequalities clearly isn’t a priority of the city government.”*	Public health opposition party
**Competences of the city government**	
*“There are many aspects that we do not deal with directly, for example the Neighbourhood Law. We manage them in collaboration with the district councils to improve housing and other structural elements.”*	Urban planning officers
*“The sewer system and subterranean lakes to prevent flooding: the city council has policies in place for these. They are partly finances by the cohesion funds*.”	Structural funds officer
**Information on health inequalities**	
*"I am not entirely sure of the causes but if you didn’t have information regarding what might be bad for your health…then you don’t know where to find it or that it is important to find it."*	Employment officer
*"Yes, we receive information from the Barcelona Public Health Agency. Within the area coordination systems, the Public Health Agency explains the principal health indicators to us every year."*	Social services officer
**Intersectoral work**	
*“Collaboration across sectors is clear to people who work in health, but it’s less clears to those working in other places.”*	Public health officer
**Collaboration with social actors**	
*“Associations also participate with us. At the beginning of its mandate the Council drafts the Strategic Action Plan and there is participation in the participatory councils. Here, in the Social Welfare municipal council, there are groups on children, poverty and elderly people’s health and they are periodically involved.”*	Social services officer
"There are territories in which neighbours and other associations are very involved and sometimes you may come across people who are willing to collaborate."	Employment officer
**BARRIERS TO POLICY IMPLEMENTATION**	
*“There are “sensitive” services which people consider need to be delivered by the council but no one wants them in their neighbourhood.”*	Employment officer
*“We come across some bureaucratic barriers. When you try and carry out a decentralised policy, then you come across opposition from the central government. When you want to implement a very innovative policy, then you have to break the inertia from the past…etc.…”*	Public health politician
**OPPORTUNITIES TO POLICY IMPLEMENTATION**	
*“When you have a law backing you up, you can defend that you have to offer services due to the law and that you do not have enough money. It is an opportunity because this makes social services universal and because it places social services in the sphere of human rights as one of the pillars of the welfare state.”*	Social services officer
*“When there is a call we submit an application but these are projects which are within the city government’s agenda. These are opportunities to accelerate what the city government already wants to implement.”*	Structural funds officer

### Health inequalities

#### Awareness and beliefs on health inequalities

Regarding the interviewees’ perceptions on health inequalities, all the informants believed there were differences in health among the city’s population. The majority of politicians perceived health inequalities as an outcome measured by differences in life expectancy and as a result of socio-economic inequalities.

***“Life expectancy is one of the most interesting indicators of inequalities; I think there was a difference of ten years between districts.”*** Environmental sector politician.

The welfare politician referred to traditional inequalities relating to material possessions along with new emerging realities associated with age, social exclusion and solitude, asylum seeking and gender. Other non-health informants explained that they perceived there were health inequalities throughout the city but did not know the exact figures. For example, the social services officer considered they were related to differences in living standards and the employment informant explained that health was associated to social cohesion and the effects neighbourhoods had on people’s health.

***“When I talk about health in neighbourhoods, I mean cohesion. In this sense people with problems make neighbourhoods problematic.”*** Employment officer.

### Causes of health inequalities

The description of the causes of health inequalities varied, making it hard to establish a clear pattern. Politicians focused mostly on socio-economic factors while officers described health related behaviours and limited resources such as income and environmental factors.

***“Air quality in Barcelona is terrible and depends where you live. There are too many pollutant particles. This is in fact an inequality.”*** Urban planning officers.

Health politicians found that health inequalities’ causes went beyond medical factors. Health officers included other individual factors such as: socioeconomic status, self-care and coping abilities, expectations regarding one’s own health and access to services, specifically health care services.

***“Socioeconomic status, cultural level, capacities to count with more health care and resources are the causes of health inequalities.”*** Healthcare services officer.

### Policies to reduce health inequalities

Politicians and all the officers described policies they were familiar with. Those from the health sector aimed specifically at health factors while politicians from other sectors mentioned policies they considered had an indirect effect on health inequalities, i.e.: traffic calming or upgrading the sewer system.

***“Everything related to the built environment is being remodelled, parks are being built, new neighbourhoods are being built…but this doesn’t affect health directly, it affects it indirectly.”*** Structural funds officer.

The health care services officer found health care policies to be the most relevant in reducing health inequalities. The health informants, both politicians and officers, felt that although health care services do address health inequalities, policies from other sectors are also necessary. The informant from the opposition party and all the health informants stressed on the importance of preventive health policies. They referred to a programme to reduce infant mortality and the “Health in the Neighbourhoods”, an intervention aimed at reducing health inequalities in deprived neighbourhoods.

***“In some services we provide health promotion, health education and prevention. We have traditionally concentrated on the old town which is the less privileged area and in the last years it has extended to a set of neighbourhoods with other indicators of deprivation by following the path of the “Neighbourhood law.”*** Public health officer*.*

### The role of the city government in reducing health inequalities

#### Priorities of the city government

All politicians and both the education and structural funds officers referred to reducing health inequalities as a clear priority for the city council. The officers from other sectors expressed that albeit not being defined as a specific objective, council policies, in general, helped to tackle health inequalities. However, health officers and the opposition party informant said that reducing health inequalities was not a priority for the current city council.

***“They aren’t the nightmare of city council members. It might be that many of them don’t think about it.”*** Public health officer.

### Competences of the city government

Most of the interviewed informants considered that action can be taken at the city level even though public health, welfare and education informants considered that many of the determinants of health inequalities are beyond the city’s authority or in the local councils within the city.

***“Many of the determinants of health inequalities go beyond city limits but there are things the city does that are beneficial for reducing social inequalities.”*** Public health politician.

The non-health officers pointed out that the city government participates in policies that are competence of the regional government but also manages the funds and contributes with financing and resources.

***“The autonomous government has the competences and puts ‘five’ towards education and the city government puts ‘three’****.”* Education officer.

According to the public health officer, the involvement of the local government in health-related matters has declined over time, a trend which would only change in the event of a major health crisis.

### Information on health inequalities

Regarding periodical information on health, the politicians brought up the Annual Health Report which is published by the Barcelona Public Health Agency and sent to them every year. The politician from the opposition party explained that there should be more discussion around the most relevant health topics in the report. All politicians remarked upon the information it provided regarding health inequalities, but the non-health officers considered there is a lack of information on health indicators and stated not receiving any information on health inequalities or other health issue.

***“I value the fact that this document is still being carried out and that it is still debated in the plenary session of the Barcelona council.”*** Opposition party informant.

Two of the health officers and politicians considered it was important to base policies on clear indicators and to evaluate them, as in some cases policies may in fact increase inequalities or have a negative impact on at-risk populations.

***No, we do not receive any information but we would like to receive it, as it would be very helpful.”*** Urban development officers.

### Intersectoral work

The politicians and officers from the health sector considered there was little intersectoral coordination in the municipal council. Most of the politicians and the officers working in urban planning referred to the Neighbourhoods Law; a participatory neighbourhood renewal programme involving intersectoral collaboration. They also explained that the council’s structure, organisation and different timescales made it difficult to collaborate with other departments. In their opinion, the other sectors were reticent to include health as a cross-cutting topic as, consequentially, other sectors would demand the same for their areas.

***“We have to restructure the way the council works so intersectoral collaboration becomes inevitable.”*** Public health politician.

The officers described examples of intersectoral work in their everyday experiences which consisted of individual collaborations between sectors for specific issues.

***“We only work with other sectors except in very specific issues because there are different territorial boundaries. Therefore social services areas don’t match health care services areas.”*** Social services officer.

### Collaboration with social actors

With regard to collaborating with social actors, all the politicians, except the opposition party’s informant, highlighted that engagement with third parties was positive. Officers gave a description of specific activities carried out by these which they considered had played an important role in helping populations at risk and addressing health inequalities. For example they helped in working with hard to reach groups and provided them with support, care and advice as well as shelter and food.

***“We have NGO’s that are playing a very important role in collecting food and basic products, a role which is unknown to society and sometimes it is even more important than the role of the public administration, and this helps to reduce theses impacts.”*** Health care services officer.

The public health political informant associated it with the work being carried out at the community level through the “Neighborhoods’ Law” programme.

***“Neighbourhood associations are a key element regarding what is being carried out in the ‘Neighbourhoods Law’.”*** Structural funds officer.

### Barriers to policy implementation

With regard to barriers they faced at work, the political informants were concerned with insufficient funds, a condition exacerbated by the economic crisis, in addition to having to compromise with factual powers or with matters lying beyond the council’s jurisdiction. The political public health, education and environmental interviewees observed how, in their experience, the opposition of some of the local population had been an important barrier and described the “not in my back-yard” effect services such as venipuncture facilities for drug users have on local residents. The employment officer also described how some groups depended solely on government benefits, which kept them from moving forward.

***“I think maybe sometimes we haven’t been brave enough to impose…”*** Education officer.

***“Sometimes, when you change streets into pedestrian zones, the shopkeepers complain an are opposed to the measure because they think their delivery van won’t be able to enter, after a while they are more than happy with the change.”*** Urban planning officers.

### Opportunities and enablers in implementing policies

Most of the officers had difficulties identifying any opportunities which had arisen while implementing a policy or intervention, and explained they had at some stage, encountered favourable outcomes they had not foreseen or planned for. For example, the social welfare politician explained that when people working in different sectors, charities or NGOs were brought together to collaborate on a specific issue, they sometimes shared information which was very helpful for all participants. The employment interviewee pointed out the high compliance of women with employability programmes. On the other hand political informants emphasized on taking confrontations and problems and turning them around by finding alternatives by combining efforts. At the same time the creation of new scenarios offered the possibility of discovering new opportunities and including these in policy making.

***“As you create these new settings, new opportunities arise, opportunities which allow to share experience and learning. To make the most of what you are doing and include it in policy making****.”* Welfare politician.

## Discussion

Policymakers identified inequalities in health among the city’s population. Politicians focused on the factors defined as structural determinants [28] as the principal causes of health inequalities, stated they had been reduced over time and counted with information on health indicators. The officers perceived individual proximal determinants as their causes, had less access to information on health indicators except when working in the health sector and focused on describing policies carried out within their sector [4]. Most of the interventions and policies were not evaluated and there was little or no intersectoral participation, only specific collaboration with social actors. The barriers described were related to bureaucracy, and the opposition of certain population groups. Opportunities described by informants made reference to collaboration between sectors or new legislation.

In our study officers and politicians referred to cultural, material, income and environmental factors as the main causes of health inequalities. Most informants were aware of the concept of health inequalities and understood that there were differences in health and life expectancy among the population. The differences in the participants’ discourses were not determined by their positions or the sector in which they worked in, contrasting with findings from similar studies in which the discourses corresponded to the latter [14,15]. In another study which described policymakers’ perceptions across Europe, also carried out within the INEQ-Cities Project [23], differences in their views was determined by the city where they worked and no pattern according to position or sector was found. In the mentioned study the sampling was opportunistic. In this study, health informants, saw health care services as important to address health inequalities, but were of the opinion that policies from other sectors were also necessary. Informants working in the non-health sectors explained that policies from their sectors as examples and considered they had an impact on reducing health inequalities.

There is a greater volume of evidence on potential interventions designed to have an impact upon individual risk behaviours [29-32]. A scoping review on policies or interventions in European cities to address health inequalities published in scientific journals, found that half of the identified papers promoted healthy behaviours [20]. Due to lack of time or simply because it may not be considered relevant to their work, officers may receive little or no further training on health inequalities and their determinants. In medical schools, in many countries, lifestyles are referred to as an individual choice. It is also highly likely that politicians work closer to matters associated with the wider determinants of health and are more aware of the causes which lie beyond individual lifestyles and this may reflect in their political views. A further study [22], also within the INEQ-Cities project, explored the perspectives of intervention managers, which in the majority of municipal structures would liaise with or have many common characteristics with the officers. They described being familiar with health inequalities and concepts such as intersectorality, participation and evidence-based action, but others such as socioeconomic aims, gradient approach, evaluation and sustainability were not so widely applied.

In a previous study performed in Canada which focused on differences between sectors regarding whether the concept of health determinants had permeated their discourse, workers in municipal governments cited ‘healthy lifestyles’ and ‘clean air and water’ as factors affecting health inequalities. ‘Strong community’ and ‘income’ were not seen as being very relevant [33]. In other studies, also carried out in Canada, which explored whether the measures applied in their fields had an impact on health outcomes, labour and social services advisors saw these as a relevant outcome while those in finance where unaware of the social determinants and their impact on health [14].

Interviewed politicians stated that reducing health inequalities was a priority for the city government and this is consistent with the reasoning that for politicians who are aware of this issue, it would be counter-productive to state otherwise. Officers’ jobs may depend less on elections and politically correct discourses and those who perform them have worked in the same sector for longer than politicians, in most cases-politicians may often change sectors after elections depending on the structure and composition of the government. It is possible that in contrast, they described a different reality: one where reducing health inequalities was not clearly set as a priority for the city council. Politicians mentioned receiving the Annual Health Report in Barcelona which is published yearly [34,35], they may be more exposed to information on health inequalities and the measures applied, and therefore, more likely to consider it a specific objective for their government than officers.

Evidence from an INEQ-Cities study which described health policy documents across European cities published around 2010 to determine how cities conceptualize health inequalities, and what strategies were applied to reduce these included Barcelona in the analysis [20]. The results showed that, for the majority of variables explored, the cities which seemed to be “doing better” were London, Stockholm and Rotterdam. The two former cities counted with examples of political leadership to address health inequalities, such as the London Health Inequalities Strategy which identified health inequalities and priorities to reduce them and a list of key partners. In the case of Stockholm, the County Public Health Policy document focused on the social health determinants throughout the social gradient [20].

There is still a need to increase the flow of evidence between research and policymaking [36] to widen the knowledge on upstream determinants and universal policies aimed at these. Similarly it has been described elsewhere [4] that policy advisors felt researchers were not able to provide them with befitting information for their policy and decision-making needs. They felt there was a lack of information on the effectiveness and cost-effectiveness of policies.

Most informants in this study referred to limited intersectoral collaboration due to the city government’s organisation and structure. However, a multidisciplinary collaboration between different sectors of the city council should be inherent to the concept of aiming policies at the social and environmental determinants of health inequalities [32,37-39]. Nevertheless, moving beyond the structural barriers in city councils may prove to be difficult and require going beyond specific individual initiatives. The lack of intersectoral collaboration is not only related to organisational barriers, but also to how health inequalities are perceived. Therefore, if-as mentioned by the policymakers in this study-there is still insufficient information made available to officers outside the health sector and a relative lack of knowledge on upstream determinants, it will be difficult to foster intersectoral collaboration. Policymakers may not be aware of the need to act upon social determinants outside the health sector. Further explanations as to why informants perceived there was little intersectoral collaboration could be related to the absence of an overarching conceptual framework and mandate such as the WHO European Healthy Cities Network model which has been taken on by some European cities, for example [31].

With regards to social actors, they were perceived by politicians as service providers rather than actual stakeholders involved in the policy making process. The officers regarded their efforts in providing services and reaching users as essential. A study [33] describing governmental and organisations workers’ perceptions described that they both agreed on having inter-institutional partnerships. The municipal governments, have smaller structures than state or regional governments and are more proximal to local institutions and citizens, and therefore, this may be advantageous for addressing health inequalities [36]. Some studies carried out in Canada described that policy advisors feared population backlash against measures implemented [12]. Although, these and other barriers described by policymakers could be overcome by liaising with community agents and introducing new measures through participatory processes which may include a diversity of stakeholders. There are successful examples of third sector involvement in addressing health inequalities in different cities throughout Europe [5]. The Neighbourhood’s Law in Barcelona [40,41], aimed at reducing social inequalities although not specifically in health, established a partnership between different sectors in the city council and community agents to renew deprived neighbourhoods.

The different historical contexts of the European countries and cities which define the course of policy making should be taken into account [32]. The Spanish democratic transition-1975-1980-has shaped health services, public policy making [34,42] and research on the determinants of health, as described elsewhere [43]. In Barcelona, the municipality was governed by a left-wing coalition during the transition and post transition periods until 2011, which probably helped to foster progressive local policies [25]. The aforementioned Neighbourhood’s Law, the Health in the Neighbourhoods Programme [44] and the social and health maternal and child intervention, implemented in a low income district to reduce infant mortality, are some examples.

### Limitations

The topic guide was sent to two interviewees who requested it so some informants might have prepared their answers and discourse. The interviewee from the environmental sector had been, in the past, a manager in the public health area, which possibly gave the informant a wider knowledge on health inequalities. There were municipal elections after the interviews, nevertheless, all the informants still work in policy related activities in the council of Barcelona and at least 9 of the 12 informants hold decision-making positions. Therefore these results may only be transferred to contexts similar to Barcelona, the results may not be the case in other locations where the political process is different.

### Strengths

We sought an informant from the opposition party to include a discourse outside the coalition government in office at the time and a more comprehensive view. Furthermore, we interviewed high profile policymakers within the municipal hierarchy selected through theoretical sampling. They defined representative informants from various sectors to be as close as possible to the saturation of the discourse, taking into account the multidisciplinary nature of health inequalities. This study has relevant methodological strengths, namely triangulation of analysts-different researchers analysed the same data-and verification of results by participants.

### Conclusions and recommendations

To our knowledge, this is among the few studies performed in Europe which reflect city government policymakers’ beliefs on health inequalities and policies to reduce them. Officers referred to specific causes of health inequalities and policies to reduce these from within their sectors and politicians clearly recognised upstream determinants of health inequalities and structural policies. Participants referred to a lack of evaluation of policies and interventions and intersectoral action was very weak. More effort should be placed on providing available information on health and its social determinants, such as the Annual Health Report and other information available along with examples of existing policies to policymakers and, in particular, to officers. Research on health inequalities and policy related issues should engage with policymakers from the early stages. Research on the multidisciplinary and multisectoral nature of tackling health inequalities should be made known to the policymakers involved, to promote health as a cross cutting issue in the city council. These should be addressed in liaison with the third sector. Some findings described may prove to be relevant not only for Barcelona, but for other cities in Europe with similar political processes and contexts. Promoting the Healthy Cities Model or strategies such as those described in London and Stockholm’s Health equity policy documents may contribute to policymakers from across the different sectors. Further efforts to monitor health inequalities should be put in place. It may also foster expanding community resources and have an impact on health.
